# Miscellany

**Published:** 1909-08

**Authors:** 


### MISCELLANY.

---

Squirrels as Plague Carriers.—The discovery in California that ground squirrels may become infected with bubonic plague is so disquieting as to be a matter of national concern, according to an editorial writer in *American Medicine* (New York, June). Says this paper:

"It is generally believed that the bacillus pestis is a normal inhabitant of some Asiatic rodent—perhaps the rat—but, from the excessive mortality among our rats, it is not likely that they are the ones with the tolerant immunity which keeps the bacillus in existence, for it is a pure parasite in nature unable to exist out of the body of some animal. So it is not at all unlikely that the ground squirrels of California now known to be infected have sufficient tolerance to keep the bacillus alive permanently in America. It will die out in time, as the cholera bacillus always does when removed from India. Yet we do know that for a long time it will be possible for the ground squirrels to infect rats of adjacent towns and start new epidemics. It is high time to take up the European demand for a world-wide war on domestic rats, which subsist almost exclusively on the wastes of human habitations. Protection or destruction of garbage and the destruction of rat nests are essentials of existence now that populations are so dense. The rats must disappear, and the only way to do it is to starve them to death. Stables are the chief culprits and it is time for a revolution in their methods of management."

